# PERSON-CENTERED DEMENTIA CARE IN SOUTH KOREA: AN INTEGRATIVE REVIEW

**DOI:** 10.1093/geroni/igad104.2414

**Published:** 2023-12-21

**Authors:** Eun-Hi Kong, Hyunju Park, Juh Shin

**Affiliations:** Gachon University, Incheon, Republic of Korea; Kangwon National University, Chunchon, Republic of Korea; George Washington University, Washington, District of Columbia, United States

## Abstract

Person-centered dementia care is a globally recognized best practice; however research on this practice is lacking. In Korea, the number of people living with dementia is rapidly increasing, expanding the need for person-centered care. Thus, we reviewed published studies regarding person-centered dementia care in Korea. We searched for studies in four databases (RISS, KISS, KoreaMED, and DBpia) and selected 14 primary studies published between 2007 and 2023. Two reviewers extracted data and three reviewers analyzed the data. Search keywords included person-centered care, person-centred care, person-centered dementia care, person-centred dementia care, dementia, demented, Alzheimer, or cognitive impairment. A total of 14 articles met inclusion criteria. The primary studies were published between 2007 and 2023, and most studies were conducted in long-term care settings and targeted formal care staff. The authors’ disciplines included nursing, social welfare, humanity, and human services. Many studies did not report theory or theoretical models. Research designs included non-experimental study (8), qualitative study (4), and experimental study (2), and randomized clinical trial (1). Many studies did not report on theory or theoretical models. Most quantitative studies included variables such as personhood in dementia, person-directed care, person-centered climate, dementia knowledge, or attitude toward dementia. Most quantitative studies reported that person-centered dementia care education programs enhanced personhood in dementia in long-term care settings. The qualitative studies explored the meaning of humanitude, readiness to adapt, effective communication, or education programs related to person-centered dementia care. Future scholars should conduct experimental studies or mixed-method studies to report on theory or theoretical models.

